# Belief, knowledge, attitude and practices towards COVID-19 amongst residents of Abuja, Nigeria: implications for pandemic preparedness

**DOI:** 10.11604/pamj.2024.47.98.34331

**Published:** 2024-03-01

**Authors:** Henry Chijioke Onyegbutulem, Dilli Dogo, Peace Ijeoma Henry-Onyegbutulem, David Samuel Olorunfemi, Peter Egbert Hermann Schwarz, Stefan Richard Bornstein

**Affiliations:** 1 Department of Internal Medicine, Faculty of Clinical Sciences, College of Health Sciences, Nile University of Nigeria, Abuja, Nigeria,; 2 Department of Internal Medicine, Nile University Teaching Hospital, Abuja, Nigeria,; 3 Department of Surgery Faculty of Clinical Sciences, College of Health Sciences, Nile University of Nigeria, Abuja, Nigeria,; 4 Department of Surgery, Nile University Teaching Hospital, Abuja, Nigeria,; 5 Department of Maxillofacial Surgery, Maitama District Hospital, Maitama, Abuja, Nigeria,; 6 Department of Maxillofacial Surgery, Ahmadu Bello University Teaching Hospital (ABUTH) Zaria, Kaduna state, Nigeria,; 7 Department of Internal Medicine, College of Health Sciences Bingham University, Bingham University Teaching Hospital, Jos Plateau State, Nigeria,; 8 Department of Medicine III, University Hospital Carl Gustav Carus, Technische Universität Dresden, Dresden, Germany,; 9 School of Cardiovascular and Metabolic Medicine and Sciences, Faculty of Life Sciences and Medicine, King´s College London, London, United Kingdom

**Keywords:** Preparedness belief, knowledge, attitude, practices, COVID-19

## Abstract

**Introduction:**

coronavirus disease, (COVID-19), was a pandemic with high global morbidity and mortality, partly due to a lack of preparedness. People´s knowledge, belief, attitude, and perception of disease outbreaks may affect their response, and this may impact their health-related behavior. This study was designed to determine the pattern of belief, knowledge, attitude, and practices (BKAP) of residents of Abuja, Nigeria, towards the COVID-19 pandemic. The outcome of the study may help to make informed decisions on future pandemic preparedness.

**Methods:**

a cross-sectional study with data collected online about the local perceptions and common concerns, beliefs, misconceptions, attitudes, and conspiracy theories amongst residents of the FCT. A self-reported validated e-questionnaire prepared on Google Forms was used. The obtained data was downloaded on Excel sheet and then exported to SPSS for analysis.

**Results:**

there were one thousand eight hundred and seventy-three (1,873) respondents, 1017 (54.3%) females and 856 (45.7%) males. Participants were majorly knowledgeable, the majority (31.2%) were in the 41-50 years age group. Surprisingly, about 17% did not know that wearing a face mask could prevent COVID-19. About 25% still met in crowded places, and slightly more than 33% did not wear outdoor masks. The highest knowledge of COVID-19 was found among people in the age range 41-50 years, females, University graduates, married people, and healthcare personnel, particularly doctors.

**Conclusion:**

our study concludes that the overall population of Abuja had good knowledge and, a positive attitude, with pockets of poor attitudes and bad practices born out of misconceptions and infodemics.

## Introduction

A pandemic, by definition, is an outbreak of infectious disease across a wide geographical area covering several continents and encompassing extensive variation in geographical landscapes, ecological networks, culture, ethnicity, and disease susceptibility among others [1]. The COVID-19 pandemic may have encouraged extensive discussions to better prevent, detect, and respond to future similar pandemics. Since the spread of a disease may be encouraged by certain cultural and ethnic dispositions and responses, it is therefore crucial to understand peoples´ beliefs. This is even more so in developing economies such as Africa´s with its health crises calling for urgent need for improved healthcare systems and pandemic preparedness. The COVID-19 pandemic, underscored the urgency for stronger epidemic intelligence and response mechanisms in case of any future occurrence. This is to prevent such embarrassment in terms of mortality and morbidity as witnessed during the pandemic [2]. The high COVID-19 transmission rate [3-5] translated to high global disease burden; morbidity and mortality [6-8], with most cases and deaths occurring in the advanced countries [6,9,10]. Thus, suggesting that the disease had the potential to overwhelm even the most advanced healthcare systems [11]. During the pandemic in Abuja, there were uncertainties and fear amidst misinformation regarding COVID-19, worsened by certain beliefs and perception with resulting attitude.

Recommendations made by the government to curtail the spread included social distancing, hand hygiene, and wearing of personal protective equipment, PPE [12,13]. As good as these measures may seem, acceptance by the people and hence effectiveness, may be affected by prevailing perceptions born out of their belief, the knowledge base of COVID-19, and their attitude towards the disease. This will also impact on their practices, some of which may be harmful [14].

It is imperative to state that, the rapid increase of cases in Abuja during the first wave of the pandemic, necessitated the lockdown of the territory by the Federal government of Nigeria. During the lockdown period, there were many unusual behaviors displayed, and misconceptions that prevailed. Available literature suggests that this kind of response during pandemics may not be new [15], and because of their significance, the WHO created a portal meant to counter negative impressions which if not checked, may affect concerted efforts to control disease spread [16].

**Objective:** the objective of this study was to examine the existing belief pattern, level of knowledge, attitude, and practices of residents of Abuja, the federal capital territory, Federal Capital Territory (FCT), towards the COVID-19 pandemic. The outcome of the study will guide further research with the hope of identifying key variables to improve preparedness to help prevent and control during future outbreaks of similar pandemics.

## Methods

**Study design:** ethical clearance with reference number FHREC/2020/01/78/17-08-20 was obtained from the FCT research and ethics committee. The study was conducted between the 15^th^ of April 2020 and the 15^th^ of May 2020.

**Setting:** this cross-sectional study was conducted on residents of Abuja, the FCT, with a population of approximately 3.3 million persons [17]. The population of Abuja is heterogenous, with a good proportion made up of civil and public servants.

**Participants:** residents who consented, could read and write English and have internet access on their phone and/or laptop devices.

**Study sample size:** the larger the target sample size, the higher the external validity and the greater the generalizability of the study [18]. Using the current population of the FCT [17], the representative target sample size needed, to achieve the study objectives and sufficient statistical power, as calculated using a sample size calculator [19], was 1,037 participants, using a margin of error of ±4%, a confidence level of 99%, a 50% response distribution. However, one thousand eight hundred and seventy-three (1,873), responses were received over a four-week period during the lockdown.

**Variables:** variables were socio-demographic characteristics which include age, sex, residential areas, occupation, level of education, and marital status. Others were; level of COVID-19 knowledge (knowledgeable, average knowledge, lack knowledge), belief type (appropriate, inappropriate), characteristics of attitude; thinks control measures are effective or in-effective, thought of COVID-19 being eradicated or not. Worries concerning COVID-19 as psychosocial assessment, and types of practices (good, bad).

**Data sources and measurements:** data was sourced solely online using social media contacts. A validated and tested questionnaire prepared on Google form was sent to social media contacts, same was rebroadcast for wider coverage. The questionnaire which was developed on Google Forms was divided into sections, assessing the socio-demographic characteristics of the study population, knowledge, beliefs, attitude, and practices sections. It was designed according to guidelines for the community of COVID-19, by the Centers for Disease Control and Prevention (CDC), [20] and the local perceptions and commonly asked questions, beliefs, and misconceptions amongst residents of Abuja at the time. The questionnaire was conducted in the English language and was validated by two independent public health experts and adjustments were made. It consisted of 1 item on consent, items 2-9 on socio-demographics including a residential area, item 10 (i-vii) on beliefs, item 11 (i-xvii) on knowledge, item 12 (i-vii) on attitude and item 13 (a-j) on practices (Annex 1). Questions on COVID-19-related misconceptions were developed based on widespread beliefs, attitudes, and practices among the people in Abuja at the beginning of the pandemic and as practiced during the lockdown. The case definition and grading system is as shown in Annex 2.

### Measurements from operational definitions

**Belief:** what is generally accepted as true concerning COVID-19. Grouped to: i) Appropriate belief; if the answer to the question is acceptable; ii) misconception or; iii) inappropriate belief if the answer is unacceptable; iv) the group was made of those who were not sure.

**Knowledge:** this is the awareness of COVID-19. It is measured and categorized as: knowledgeable (70-100%/22.4-32 points), average knowledge (50-69%/16-22.3 points), lack of knowledge (< 50%/<16 points).

**Attitude:** the way people think and behave towards COVID-19. It is measured by 11 questions. The percentage of persons with different attitudes were computed and classified into good attitudes and bad attitudes.

**Practice:** habitual involvement to prevent COVID-19. It is measured by 5 questions. The percentage of persons with different practices were computed and classified in to: good practice, bad practice.

**Bias:** people who could not read or write in English language, and those who did not have internet access were omitted. This was however a small group, considering the high percentage of educated persons in Abuja, which is highly populated by politicians, professionals, public and civil servants. Another potential bias was using my social capital i.e. those with known social media contacts. However, effort was made to counter this by encouraging rebroadcast by the primary contacts.

**Quantitative variables:** quantitative variables represented in numbers/percentages include age range, and number of persons per household. Qualitative variables that were converted to quantitative and represented as percentages include belief, knowledge, attitude and practices. Gender, occupation, marital status, and nationality.

**Statistical methods:** data from Google Forms was downloaded on an Excel sheet and then exported to SPSS for analysis. Analysis was done using STATA version 15 (Stata Corp, USA). The variables were presented as percentages/frequencies. Pearson Chi-square (cross-checked using Fisher´s exact test on some occasions) was used to test for associations between COVID-19 knowledge base and age range, sex, level of education, occupation as well as marital status. A p-value of < 0.05 was set as significant.

## Results

**Participants:** one thousand eight hundred and seventy-three (1873), respondents consented to and participated in the online survey by completing the questionnaire. There were 1017 (54.3%) females and 856 (45.7%) males. Most of the participants (31.18%) were in the 41-50-year age group. Table 1 displays the socio-demographic characteristics of the participants. They were mostly Nigerians, married, and university graduates. Table 2 shows the distribution of the frequencies of the various belief items. The commonest misconception was that COVID-19 was a biological warfare, followed by the belief that ingestion of hot fluids and certain herbs, will prevent and cure COVID-19. No one believed that COVID-19 could be transmitted by witchcraft attacks. Table 3 displays the basic knowledge level of the Abuja residents of COVID-19. They were quite knowledgeable (average score of 90%). Surprisingly, about 17% did not know that wearing a face mask could prevent COVID-19. Table 4 represents the attitude. More than 60% percent felt that the measures put in place by the government were not sufficient to prevent COVID-19 spread. Also, 18.6% rejected the possibility that family members could contract COVID-19. Less than 40% were worried about the prevailing circumstance, less than 25% were disturbed, and not up to 20% were worried about the risk to their health or that of their family members.

**Table 1:** socio-demographic characteristics of participants who took the survey in Abuja between the 15^th^ of April and the 15^th^ of May 2020

Variables	Value (%)	Variables	Value (%)
Age range (n)		Marital status (n)	
10-20 years (57)	3.04	Married (1220)	65.14
21-30 years (463)	24.72	Single (580)	30.97
31-40 years (493)	26.32	Widow (30)	1.6
41-50 years (584)	31.18	Widower (28)	1.49
51-60 years (262)	13.99	Separated (15)	0.8
> 60 (14)	0.75	**Level of education (n)**	
**Sex (n)**		Secondary school (14)	0.75
Male (856)	45.7	High school (28)	1.49
Female (1017)	54.3	First degree (1081)	57.71
**Religion (n)**		Post graduate (750)	40.04
Christianity (1450)	77.42	**Number per household**	
Islam (422)	22.53	One (118)	6.3
Others (1)	0.05	Two (203)	10.84
**Nationalities (n)**		Three (113)	6.03
Nigerian (1845)	98.51	Four (223)	11.91
Other Africans (27)	1.44	Five (464)	24.77
Non-Africans (1)	0.05	Six (303)	16.18
		Seven (147)	7.85
		Eight (58)	3.1
		Nine (87)	4.64
		Ten (72)	3.84
		Eleven 71()	3.79
		Thirteen (14)	0.75

**Table 2:** frequencies of appropriate beliefs and misconceptions amongst participants who took the survey in Abuja from the 15^th^ of April and the 15^th^ of May 2020

	Belief Item	Appropriate (n)%	Inappropriate (n)%	May be (n)%
1	5G is a cause of COVID 19	1756 (93.75)	117 (6.25)	0(0.00)
2	COVID-19 is a biological weapon	10433 (55.69)	830 (44.31)	0(0.00)
3	COVID 19 is a curse	1247 (66.58)	116 (6.19)	510 (27.23)
4	COVID-19 is transmitted via witchcraft	1873 (100)	0 (0.00)	0 (0.00)
5	COVID-19 can be cured by the Native	1843 (98.40)	30 (1.60)	0 (0.00)
6	Alcohol ingestion is useful in prevention and treatment	1786 (95.36)	87 (4.64)	0 (0.00)
7	Ingestion of hot tea is useful in prevention and treatment	1333 (71.17)	540 (28.83)	0 (0.00)
	Average	83.00%	13.00%	4.00%

**Table 3:** knowledge levels amongst participants who took the survey in Abuja from the 15^th^ of April and the 15^th^ of May 2020

		Knowledge		
	Questions	Knowledgeable	Average knowledge	Lack knowledge
1	Alcohol-based hand sanitizers can denature or arrest the virus	1495 (79.82)	335 (17.89)	43 (2.30)
2	Key clinical symptoms are fever, fatigue, dry cough, breathlessness and muscle pain	1758 (93.86)	87 (4.64)	28 (1.49)
3	Unlike the common cold, stuffy and runny nose, are less	1366 (72.93)	392 (20.93)	115 (6.14)
4	Recent travels to a country with COVID problems and is sick with fever should have a COVID test	1800 (96.10)	14 (0.75)	59 (3.15)
5	In the early phase, CORVID 19 may look like Malaria	1398 (74.64)	388 (20.72)	87 (4.64)
6	Currently, there is no standard/effective cure, but early symptomatic/supportive treatment can help	1844 (98.45)	14 (0.75)	15 (0.80)
7	Not all persons with coronavirus will develop severe form	1714 (91.51)	144 (7.69)	15 (0.80)
8	Severe forms of the disease may occur in the elderly, people with chronic diseases	1815 (96.90)	44 (2.35)	14 (0.75)
9	Unless fever is present, people with COVID-19 cannot infect others	1511 (80.67)	334 (17.83)	28 (1.49)
10	COVID-19 virus spreads via respiratory droplets of infected persons	1845 (98.51)	28 (1.49)	0 (0.00)
11	General medical masks can help prevent COVID-19 infection	1452 (77.52)	101 (5.39)	320 (17.08)
12	It is not necessary for children and young adults to take measures to prevent the infection	1656 (88.41)	15 (0.80)	202 (10.78)
13	To prevent the COVID-19 infection, individuals should avoid going to crowded places	1873 (100)	0 (0.00)	0 (0.00)
14	Isolation and treatment of infected people are effective ways to reduce the spread	1873 (100)	0 (0.00)	0 (0.00)
15	Contacts of COVID-19-positive persons should be immediately isolated	1873 (100)	0 (0.00)	0 (0.00)
16	The usual duration for isolation is .......days	1713 (91.46)	146 (7.79)	14 (0.75)
	Average	90%	7.00%	3.00%

**Table 4:** attitudes of study participants

	Participants	Number (%)	Number (%)	Number (%)
1	How effective are social distancing measures to slow spread of COVID-19	Effective 1614 (90.87)	Ineffective 99 (5.23)	Don’t know 73 (3.90)
2	Nervous and worried about current situation of my health and that of my family	Yes 1435 (76.62)	No 14 (0.74)	Indifferent 424 (22.64)
3	How likely is it that someone from your family will be getting COVID-19	Likely 276 (14.74)	Unlikely 1249 (68.1)	Don’t Know 348(18.57)
4	On the response of the Nigerian Government	Sufficient 1275 (68.07)	Insufficient 541 (28.88)	Don’t Know 57 (3.04)
5	How worried are you about the effects of COVID-19 on your household's economic situation?	Worried 1744 (93.111)	Not worried 129 (6.89)	Indifferent 0 (0.00)
6	COVID-19 will be successfully controlled with all what the government has in place	Yes 1641 (87.61)	No 29 (1.55)	Don’t know 203 (10.84)

Table 5 summarizes the common practices towards COVID-19 during the lockdown as the disease incidence surged in the first wave. The practices were not quite commendable. Above 25% still met in crowded places, and slightly more than 33% did not wear outdoor nose masks. A reasonable percentage still went out some in social gatherings, Reasons for going out include buying food for self/family (64.4%), doing exercise (31%), going to work as essential duty staff (18.8%), tired and bored staying indoors (14.6%) among others. About 24% took boiled concoctions mix of ginger, garlic, and lemon in different proportions, while about 23% had regular hot beverages or hot baths (13%).

**Table 5:** frequencies of good and bad practices of the study participants

			Good	Bad
			n (%)	n (%)
1	Have you gone to crowded places recently		No1394(74.43)	Yes 479 (25.57)
2	Do you wear facemask always when leaving the house?		Yes1247(66.58)	No 626 (33.42)
3	I stay at home		Yes 667 (35.6)	No 1206(64.4)
4	I did not attend social gatherings		Yes 422 (22.5)	No 1451(77.5)
5	I ensured social distancing always		Yes 1339 (71.5)	No 534 (28.5)
6	I wash my hands more often now than before		Yes 1566(83.6)	No 307 (16.4)
7	If I have symptoms of sickness, I will immediately inform those around me		Yes 1859 (99.3)	No 14 (0.7)
8	Have been consuming Lemon to prevent COVID-19		No1421(75.87)	Yes 452(24.13)
9	Have been consuming ginger and garlic to prevent COVID-19		No1408(75.17)	Yes 465(24.83)
10	I take hot baths to prevent COVID-19		No1611(86.01)	Yes 262(13.99)
11	I now take some alternative medicines and Roots to prevent COVID-19		No1758(93.86)	Yes 115(6.14)
12	I consume alcoholic beverages to prevent COVID-19		No1815(96.90)	Yes 58(3.10)
13	I consume hot tea beverages to prevent COVID-19		No1436(76.69)	Yes 437(23.33)
	Average		73.70%	26.30%
	**Reasons for going out by participants during the lockdown n (%)**			
1	Tired of staying indoors 273 (10.8%)			
2	Doing exercise (22.93%)	580		
3	Meeting friends and relatives 14 (0.55%)			
4	Buying food for myself and my family 1207 (47, 73%)			
5	I am on a daily pay job and must go to get my daily bread 14 (0.55%)			
6	Going to work as essential duty staff 352 (13.92%)			
7	Had an emergency 74 (2.93%)			
8	To get a particular service I needed badly 15 (0.59%)			
				

Table 6 shows associations between the knowledge base of COVID-19 and some variables, age range, sex, level of education, occupation, and marital status.

**Table 6:** associations with knowledge of COVID-19 amongst participants of the study and reasons for going out by participants during the lockdown

	Pearson-Chi^2^ test	Fisher’s exact test	p-value
Age (41-50) years	52.6		0.000
Sex (female)	11.16	0.001	0.001
First degree	53.37	0.000	0.000
Doctor	607.7		0.000
Married	91.25	0.000	0.000

## Discussion

Being the most populous African country and the seventh most populated country globally, Nigeria´s population was at risk for high COVID-related morbidity and possibly mortality. Although this potential existed, factors that may have prevented massive deaths in this tropical country, as experienced in the temperate regions need to be ascertained.

**Level of education:** our study population was an educated one, with the majority being females. This female dominance in taking BKAP assessments towards COVID-19 is consistent with earlier reports from China and Jordan [21,22], but not so with previous studies involving Nigerian subjects [23,24]. Coincidentally, available literature suggests a better female gender health-seeking behavior [25,26], a piece of vital information that may be helpful when planning future targeted preparedness campaigns. Interestingly, the previous Nigerian studies were carried out in the northern parts of the country with levels of social exposure skewed towards the male gender, possibly for religious reasons. This high percentage of educated persons in this study who again were mostly married, (representing households) (Table 1), carries an advantage of better knowledge base, and the fact that empowerment will be easier with better coverage since more households rather than individuals will be reached. This has a positive impact on preparedness even for the future. Earlier reports from Asia [21,27-29], and other parts of Africa aside from Nigeria [30-32], indicated high COVID-19 knowledge among the studied populations, which reflected the caliber of respondents with commendable level of education as with our study population.

**Role of age and social media:** the role of social media, (mostly utilized by educated persons), in pandemic-related information gathering and dissemination, cannot be overemphasized [21,29,30]. As a fact, educated and young people constitute the biggest users of social media in Nigeria [33]. Most of the participants in this study were within the young age bracket. From when the WHO declared COVID-19 a pandemic, several guidelines and information, who were uploaded online by the WHO, and such information was easily accessible by internet users in Nigeria, who were mostly young people. Access to such reliable information could help dispel inappropriate beliefs, misinformation, and misconceptions and may help curtail spread during pandemics.

**COVID-19 knowledge base:** specifically, our respondents were very knowledgeable towards COVID-19 (Table 3). There was an average of 90% correct rate in the COVID-19-related knowledge questions around the symptoms, mode of transmission, and preventive measures. Interestingly, similar-online-based studies within Nigeria, Africa, and beyond revealed a high knowledge base of respondents [21,24,27,34,35]. This may be because, at that time, people were very curious on knowing much about this novel disease. Also, various governments embarked on educating their populations about the disease. The case with Nigeria was a very commendable effort of the Federal government through the Presidential task force on COVID-19, in educating people through the media, including online sources about the disease. Those who could not read or write as well as those without internet access were excluded from our study. Opinion from these groups may be sorted independently for comparison purposes.

Knowledge of COVID-19 was limited in some areas though. Here, some matters of concern include failure to recognize some early COVID-19 features, especially the nasal symptoms in up to 21% of persons. Close to that number failed to note that, COVID-19 in its early stages could mimic malaria. About 18% did not know that not all COVID-19 cases will present with obvious fever initially, and so these asymptomatic patients could transmit the disease.

In a very recent study by Onyegbutulem *et al*. in Abuja, only 15.2% of confirmed COVID-19 cases had positive travel history at the time when COVID-19 was still believed to be imported from Europe and America [36]. Furthermore, only 36.7% of cases had traceable contacts with known confirmed COVID-19 index cases [36]. This suggests that a good number of COVID-19 infections may have been contracted from asymptomatic or pre-symptomatic subjects through community spread, which was dreaded during the first wave. Interestingly, there are existing reports of COVID-19 transmission by asymptomatic and pre-symptomatic patients [37-39]. Epidemiologically, this is serious as it may have a negative impact on the fight against a pandemic since seamless community spread may be a routine [40]. This kind of “hidden spread” may have been a distracting factor on some occasions. For example, initially, despite the rising number of cases in Lagos, there were perceived few cases within Abuja [41], leading residents to believe they were at lower risk of contracting the COVID-19 virus, a pattern that was reported in a study conducted among residents in some districts of China that eventually became the global epicenter for the pandemic [21]. This deceptive picture allowed unconcerned behavior, with snowballing of the number of cases in Abuja which subsequently became the second epicenter of COVID-19 cases in Nigeria, after the Lagos.

About 5.4% and 17% respectively, of our study subjects were either not sure, or did not know that face masks could prevent COVID-19, (Table 3) (knowledge). Even more worrisome was that, this lack of knowledge may have significantly influenced practices towards mask-wearing. This was displayed by the finding that 33.42% of our respondents did not wear face masks routinely when leaving the house (Table 3). Although this study did not examine specifically which groups were more unlikely to wear masks as a routine COVID-19 preventive measure, previous studies have related this bad practice to a low level of education with some believing that COVID-19 was a disease of the rich and those in the high social class who travelled abroad to contract the disease [23,24,42]. This may not have been a major reason in our study subjects who were mainly educated. Some complained of the discomfort associated with wearing masks for a long time, which may be a discouraging factor.

**Belief and misconceptions:** in this study, the belief was on the overall appropriate, 83%, reflecting a good measure of preparedness at the time of the pandemic. Therefore, there was a 17% misconception rate; mainly from the belief that COVID-19 was a biological weapon attack, (43.3% of the 17%) (Table 2). A report from Iran [43] stirred up this conspiracy theory of COVID-19 being a weaponized virus with the widespread misconception that followed. This misconception was reported in an earlier Nigerian study by Olapegba *et al*. [32] with a slightly lower rate than we found (43.3% vs 47%). This had the potential of distracting most of the population to resort to other measures such as prayers and religious rituals during such disease outbreaks with little or no attention to laid down infection prevention and control measures [44]. If not corrected through the dissemination of factual information, such misconception may affect control measures now and subsequently with a negative impact on preparedness.

The theory that the 5G network was the cause of COVID-19-related disease, as a serious misconception, was also accepted in Abuja, Nigeria. However, the acceptance rate was slightly less than the 7.3% found in a study sounding the entire sub-Saharan Africa region through an online data-sourcing methods too [45]. Proponents of this conspiracy theory believed that the electromagnetic radiation from the 5G technology was responsible for the mutation of the coronavirus; and that the 5G technology alongside a weaponized virus were strategies set to control the population of the less industrialized nations among others [46-48]. This is because radiofrequency radiation (RF) was increasingly being identified as a new form of environmental pollutant [47], more so when the disease showed up only shortly after the deployment of the 5G network in Wuhan, China [49].

The other misconception of note was the ingestion of hot tea to cure or prevent COVID-19 infection (Table 2). This also doubled as an inappropriate practice. This was widely believed in and practiced (Table 5) during the lockdown in Abuja. However, its effectiveness has not been scientifically proven [50].

About 6.2% of participants inappropriately considered the disease a result of a divine curse, and a good number (27.23%) were undecided if COVID-19 was due to a curse from God. This undecided group had a high chance of being swayed to wrongly believe so, given the high level of religiosity amongst Nigerians [44]. The direct involvement of religious leaders and clerics by the Nigerian government in the fight against COVID-19 and similar pandemics would immeasurably aid in the dissemination of information about this and similar diseases.

**Attitude:** optimism is very vital in limiting disease spread during pandemics and possibly prevention of future pandemics as that represents a positive attitude. The establishment of early alerts and global warnings should be considered key in preventing the occurrence of a global pandemic in the future. Our study shows that, there was a positive attitude, and that COVID-19 will finally be controlled (Table 4). This was clearly displayed by the high optimism about the effectiveness of the social distance measures (90.87%), individual concerns over personal health and that of family members (76.62%), trust that the efforts by the government will be fruitful (87.61%), concerns over the economic situation of their households (93.11%) and thus not wanting self or family members to contract COVID-19 (68.1%). A similar positive attitude was reported in earlier studies from Nigeria and elsewhere [21,24,27,34,35], depicting a common attribute of a desire to see the end of the pandemic which had impacted so much on individual and collective psychological, social, and economic lives with severe strain on other aspects of medical care [51]. This gives a good indication of preparedness to fight the pandemic and even future ones. An earlier study from Nigeria expressed concern over a lower rate of confidence in government effort of 71% [24] when compared with previous studies from elsewhere that showed confidence rates of between 84% and 97% [21,27,34,35].

Our own study revealed a confidence rate of 87.61%, which was also higher than that in the previous Nigerian study which took place in Katsina, in the far northern parts of Nigeria. The previous studies from Asia [21,27] attributed their high confidence rates to precedence set by their governments´ successful efforts in curbing previous pandemics, and so were certain that the previous successes would be replicated. This again is another veritable fact that perception today may impact on perception in the future with a direct impact on the level of preparedness and hence reduce disease spread. We had observed earlier that, the study from Katsina, Northern Nigeria, was the only one among the referenced studies with male dominance. Whether this had an impact on the outcome remains to be further ascertained. Also, Katsina is not as metropolitan as Abuja and so the uptake of the government´s empowerment information programs may have been limited with the potential to undermine the fight against COVID-19.

**Practices:** the average percentages of the overall good and bad practices are shown in Table 5. The level of good practices was not particularly impressive as would have been expected, given the high knowledge base and level of education, of the study respondents. The social distance-related questions 1 to 5 of the practices questionnaires of Table 5, revealed bad practice rates of; 25.57% (went into crowded places including markets and worship centers), 64.4% (did not stay at home), 77.5% (attended social gatherings) respectively. Social distancing has long been recognized as a very important practice towards control of pandemics [52]. This was not followed to a good measure, which could have partly explained why FCT was the second epicenter in Nigeria. Common reasons for going out (Table 5) include buying foodstuff (47.73%), doing exercise (22.93%), and going to work as an essential duty staff (13.92%) amongst others. Recall the high level of education among our respondents, and Abuja being partly a civil/public service city, a good number had to go to work, especially the senior staff cadre, level 14 and above, where most of our respondents likely belonged. The earlier study in Katsina, Nigeria, had more men as respondents. Men were more likely to have attended crowded places, and that study stated that most of the men were mainly physical laborers, self-employed persons, small business owners as well as those who attended to religious obligations.

Certain practices without proven scientific efficacy were adopted especially during the first wave. These include consuming hot lemon juice (24.13%), consuming hot ginger/garlic juice mixture (24.83%), taking hot baths (13.99%), as well as ingesting hot beverages (23.33%) and ingestion of alcohol (3.10%). Most of the people who ingested consumable alcohol including, locally brewed spirits, gin, rum, whiskeys, etc. for this purpose, did so as a bad practice in response to the misconception that, since hand rub alcohol is said to be useful in hand hygiene, they reasoned that consumable alcohol may be useful for “throat sanitization” since the virus invades through the upper airway. While alcohol (at a concentration of at least 60% by volume, which is dangerous for human consumption), works as a disinfectant on the skin, it has no such effect within the system when ingested, it is toxic, and besides, the virus lodges in the hard-to-reach area of the nasopharynx [53]. Thus, ingestion of alcohol will not prevent or treat COVID-19 infection. This indicates that despite possessing significant knowledge about COVID-19 by most of the respondents and being well educated as earlier reported, the respondents were still largely influenced by misinformation and misconception, and certain beliefs. Proven methods should be propagated as part of the strategies of the fight against COVID-19 and part of future preparedness. Elsewhere, there are documented suggestions that traditional medicine from homeopathy and ayurveda may fight COVID-19, and close to a third of the population practiced that [54].

One preventive measure that was widely practiced was hand hygiene, by about 84% of our subjects. High rates of this useful practice were reported globally in previous studies [21,24,27,34,35,43]. An impressive 99.3% of our subjects reported that if they developed symptoms suggesting COVID-19, they would inform those around them, and of course, take a test. This would give an opportunity for self-isolation to break the disease chain if the test result returned positive.

This study showed interesting associations between knowledge of COVID-19 and certain variables as displayed in Table 6. The age range 41-50 years showed a strong association, p=0.000, with a good knowledge base for COVID-19. This age range was higher than the age from an earlier report from Nigeria that involved undergraduate university students [55] who also had a good COVID-19 knowledge base. That same study showed a better knowledge-score among males than females. This gender difference was inconsistent with our finding which showed a strong association between high COVID-19-knowledge and female gender, consistent with the report by Lee *et al*. [56]. In our study, having a higher level of education, particularly first degree was positively associated with high knowledge of COVID-19, consistent with an earlier finding by Lee *et al*. [56]. The study by Lee *et al*.did not find a significant association between knowledge of COVID-19 and marital status, socio-economic status, and area of residence, as opposed to ours. These differences may be due to disparity in socio-demographic factors.

That said, it is therefore necessary to know the existing level of knowledge of Abuja residents about COVID-19, their belief profile and misconceptions, attitude towards the disease, as well as adopted practices. Such data is necessary for the promotion of major preventive behaviors and interventions as well as appraising emanating challenges now and possibly for future eventualities.

**Limitations and strengths:** limitations of this study include passive exclusion of people who could neither read nor write in English; the survey was restricted to people with internet access. Although the high level of COVID-19 awareness among the respondents signified a positive predictor in curtailing the COVID-19 pandemic and a veritable reflection of readiness towards future similar pandemics, the exclusion of the underprivileged individuals, whose status may indeed fuel the spread of the disease leaves a gap. However, this group constitutes a small proportion of the Abuja population. The strength of the study is the large sample size.

**Recommendations:** the government should create tools to increase awareness and adoption of safe practices through the many available media outlets which are simple ways of reaching out. A strong system that provides sustained campaigns to debunk COVID-19-related misinformation and disinformation should be encouraged. This finding is very critical for the future of Nigeria´s fight against COVID-19 or similar pandemics.

## Conclusion

Our study concludes that the overall population of Abuja had acceptable knowledge, a positive attitude, and adopted certain practices to prevent and treat the disease during the pandemic. There were, however, unimpressive pockets of misconceptions, poor attitudes, and bad practices born out of misconceptions and infodemics, a situation that may counter preventive efforts now and preparedness for the future.

### What is known about this topic

- *Belief about and knowledge of COVID-19 influenced people´s attitudes and practices towards the disease*;
- *Misconceptions existed during the COVID-19 pandemic*.

### What this study adds

- *Surprisingly, a good number of people despite their level of education, did not know that wearing of face mask could prevent COVID-19*;
- *Females, married people, university graduates, and people in the age range of 41-50 years have the highest knowledge of COVID-19 in Abuja, Nigeria*.

## Annexes

**Annex 1**: questionnaire (PDF-144 KB)

**Annex 2**: grading/classification of belief, knowledge, attitude, and practices of the study group (PDF-84 KB)
